# Factors affecting home environmental safety management for fall prevention for older adults in northern Thailand

**DOI:** 10.1186/s12877-023-04419-7

**Published:** 2023-10-31

**Authors:** Archin Songthap, Pattama Suphunnakul, Jutarat Rakprasit

**Affiliations:** Faculty of Public Health, Naresuan University, 99 Village 9, Thapho Subdistrict, Muang District, Phitsanulok, 65000 Phitsanulok Province Thailand

**Keywords:** Home environmental safety management, Fall, Prevention, Older adults

## Abstract

**Background:**

Falls among older adults affect physical and mental health, disability, death, and quality of life. Home environmental safety management helps to reduce the risk of falls among older adults. This analytical cross-sectional study aimed to determine factors affecting home environmental safety management for fall prevention for older adults in northern Thailand.

**Methods:**

The study sample included 328 relatives who looked after older adults in their families in Phitsanulok Province, northern Thailand. They were randomly selected by a multistage sampling technique. Data were collected by a self-administered questionnaire consisting of 7 parts: (1) sociodemographic characteristics, (2) knowledge about home environmental safety management, (3) perceived susceptibility of falls, (4) perceived severity of falls among older adults, (5) perceived self-efficacy of home environmental safety management, (6) perceived outcome of home environmental safety management, and (7) home environmental safety management. Frequencies, percentages, means, standard deviations, and multiple regression analysis were employed for data analysis.

**Results:**

The majority of participants (60.4%) had high scores for home environmental safety management for fall prevention for older adults (scores of 14–20). Factors that significantly affected home environmental safety management included perceived severity of falls among older adults (β = 0.323), perceived self-efficacy of home environmental safety management (β = 0.311), the elderly family member having fallen in the past year (β = 0.217), being a grandchild of an older adult (β = -0.143), perceived outcome of home environmental safety management (β = 0.142), and being widowed, divorced or separated (β = -0.096). These 6 factors explained 35.1% of home environmental safety management for fall prevention for older adults.

**Conclusion:**

Relatives who look after older adults should be educated about the perceived severity of falls among older adults, perceived self-efficacy, and perceived outcome of home environmental safety management. The focus should be on grandchildren of older adults and those who are widowed, divorced or separated to understand how home environmental safety management is important to prevent falls and their consequences among older adults.

## Background

Thailand transitioned from an aging society to a completely aged society in 2022, and the proportion of older adults is greater than 20% of the whole population [1]. Being an aging society reflects changes in population structure in both urban and rural areas. Therefore, it is crucial to understand the physical changes that occur with aging to prepare for an aging population. These health conditions impact safety in the home environment. Appropriate home environmental management can prevent hazards and injuries in older adults. It also helps older adults perform daily activities on their own when they are alone. Furthermore, home environment safety can reduce the risk of slipping and accidental falls [2].

Accidental falls among older adults cause disability from hip fracture and head injury and are the main cause of death among older adults. Most hip fractures in older adults result from osteoporosis [3]. Falls among older adults lead to damage, deterioration, and harm to other organs, such as fractures and head injuries [4]. Additionally, most older adults have health conditions such as hypertension, diabetes, heart diseases, and stroke. These health problems contribute to increased complications and risk of falls among older adults [5].

In 2019, 24,364 older adults who experienced falls received emergency medical care, including 24,364 males and 10,745 females. The proportion of older adults who received emergency medical care resulting from accidental falls increased by 29.5% from 2016 to 2019. Most of the injuries occurred in older adults aged 60–64, 65–69, and 70–74 years. There were more cases among males than among females. However, more cases among females than among males have been reported among those aged 70 years and over [6]. Moreover, the death rate caused by falls potentially increased between 2017 and 2019 (9.0, 10.2, and 11.7 per 100,000 population, respectively). The number of deaths among males was 3.5 times higher than that among females, whereas the number of falls among females was 1.6 times higher than that among males. Approximately 65% of falls among older adults took place in the home area. The cause of falls in the home area resulting from slipping and tripping or taking wrong steps accounted for 65.4%, whereas 5.6% of falls were caused by falling from stairs [7].

The Department of Disease Control, Ministry of Public Health, Thailand (2021) [8] reported that the incidence of falls among older adults was approximately 3 million cases per year, and approximately 60,000 patients were hospitalized. In addition, 4 patients died per day, and accidental falls among older adults often occurred in the rainy season. However, there is no report of the incidence or prevalence of falls among older adults in northern Thailand.

Previous studies revealed that factors affecting home environmental safety management for fall prevention for older adults include personal characteristics such as sex, age, marital status, occupation, income, and education level [9, 10]. Physical impairment, health condition, and having experienced falls in the past also cause falls among older adults [11]. Moreover, type of dwelling, knowledge of fall prevention, perceived susceptibility of falls, perceived severity of falls, perceived benefits, perceived barriers, perceived self-efficacy of home environmental safety management, and perceived outcome of fall prevention are related to home environmental safety management [10, 12–14].

Some older adults experience physical deterioration and health conditions. Falls among older adults affect their physical and mental health. Some of them become disabled and bedbound because of severe falls [5]. Falls among older adults increase expenditures that families must deal with, such as for health care services [15]. Moreover, older adults who have health conditions and complications can have an increased risk of death [16].

Although some previous studies have assessed factors influencing home environmental safety management for fall prevention among older adults, there is little information about this topic in northern Thailand. Our study applied protection motivation theory to determine factors affecting home environmental safety management for fall prevention for older adults. The theory describes how influences on individuals’ perceptions of health problems can result in changing behaviors to prevent falls among older adults. Our study collected data from relatives of older adults since they play a key role in decision-making for the daily lives and health care of older adults in Thai culture. The results of this study will be utilized for creating home environmental safety management programs to reduce accidental falls, disability, and death and improve the quality of life of older adults.

## Methods

### Study design, setting and participants

This was an analytical cross-sectional study. The population was relatives who looked after older adults in the family in Phitsanulok Province, northern Thailand. The sample included 328 relatives of older adults consisting of children, grandchildren, sisters or brothers of older adults. The sample size was calculated using the estimation of the population mean [17] based on 2,230 people, and the variance (σ^2^) of home environmental safety management was 0.3 [18]. We set the error of estimation (e) at 0.03, and the alpha (α) was 0.05. The inclusion criteria included (1) age 20 years or over, (2) being able to write and read Thai, (3) at least 1 year of caring for older adults, and (4) willingness to participate in this study. The exclusion criteria included (1) sudden illness on the date of data collection and (2) inability to complete the questionnaire. A multistage random sampling technique was utilized to recruit participants. One district out of 9 from Phitsanulok Province was selected as a representative using a simple random sampling technique. Then, 7 subdistricts followed by 7 villages out of 10 were selected by a simple random sampling technique. Finally, the participants from each village were randomly selected using the same technique.

### Research tool

Data were obtained by a self-administered questionnaire consisting of 7 parts: (1) sociodemographic characteristics, (2) knowledge about home environmental safety management, (3) perceived susceptibility to falls among older adults, (4) perceived severity of falls among older adults, (5) perceived self-efficacy of home environmental safety management, (6) perceived outcome of home environmental safety management, and (7) home environmental safety management. Sociodemographic characteristics included 13 items regarding data on older adults and on the relatives who looked after them. Knowledge was assessed through 10 yes-no questions and classified into 3 categories using a sum score [19]: good (scores of 8–10), average (scores of 6–7), and low (scores of 0–5). The perceived susceptibility, perceived severity, and perceived outcome items consisted of 10 5-level Likert scale questions starting from strongly agree to strongly disagree and classified into 3 groups [20] using mean scores for high (3.68–5.00), average (2.34–3.67) and low (1.00–2.33). Perceived self-efficacy involved 10 5-rating scale questions ranging from strongly believe to strongly disbelieve and was divided into 3 groups using mean scores for high (3.68–5.00), average (2.34–3.67) and low (1.00–2.33). Home environmental safety management had 20 yes-no questions and was grouped into 3 categories using sum scores for good (14–20 scores), average (7–13), and low (0–6). All questions with an item objective congruence (IOC) index greater than 0.5 were considered to meet the standard criteria of the validity test. The reliability of the questionnaire was evaluated among 30 respondents who were not included in this study. The reliability test yielded Cronbach’s alpha coefficients, except for knowledge, which was assessed by the Kuder-Richardson 20 (KR-20). The reliability scores of knowledge, perceived susceptibility, perceived severity, perceived self-efficacy, perceived outcome, and home environmental safety management were 0.74, 0.89, 0.78, 0.91, 0.84, and 0.76, respectively.

### Data collection

This study was carried out between March and June 2022. Data were separately collected at the 7 selected villages using a self-administered questionnaire. Participants who met the inclusion criteria and were willing to participate in this study were requested to complete the questionnaire. Participants were spaciously seated to maximize confidentiality and to minimize possible contamination of information due to peer discussions. Responding to the questionnaire took approximately 30 min. Then, all complete questionnaires were used for data analysis.

### Statistical analysis

Data were analyzed using a statistical software program. Descriptive statistics, including frequencies, percentages, means and standard deviations, were used to describe sociodemographic characteristics, knowledge, perceived susceptibility, perceived severity, perceived self-efficacy, perceived outcome, and home environmental safety management. Stepwise multiple regression analysis was employed to determine factors that affected home environmental safety management for fall prevention for older adults. All significance levels were set at 0.05.

## Results

### Sociodemographic characteristics of participants and older adults

Of 328 participants, 66.3% were female, and 34.8% were aged 40–49 years (mean = 45.25, S.D.= 13.52). Approximately two-thirds (66.5%) were married, and 44.6% had an average monthly income of 5,000–9,999 Thai baht (mean = 6,671.07, S.D. = 4,129.48). Slightly more than one-third (34.1%) of participants had finished high school or an equivalent, and 42.1% were employees. The majority of participants (38.4%) had spent less than 5 years taking care of older adults (mean = 10.92, S.D. = 10.20), and 71.1% were children of older adults. Most of the older adults (40.9%) who were under care were aged 70–79 years (mean = 72.32 years, S.D. = 7.87). Approximately 60% of older adults had chronic health conditions, 15.2% had physical impairments, 17.7% had a history of falls in the past year, and 42.4% lived in a 2-floor house (Table 1).

**Table 1:** Sociodemographic characteristics of participants and older adults (n = 328)

Variables	n (%)	Variables	n (%)
**Sex**		**Average monthly income (Thai baht)**	
Male	117 (35.7)	<5,000	146 (44.5)
Female	211 (64.3)	5,000–9,999	153 (46.6)
**Age (years)**		≥10,000	29 (8.9)
<30	39 (11.9)	(Mean = 6,671.07, S.D.=4,129.48)	
30–39	64 (18.3)	**Time spent caring for older adults (years)**	
40–49	114 (34.8)	≤5	233 (38.4)
50–59	69 (21.0)	6–10	51 (28.4)
≥60	46 (14.0)	>10	44 (32.2)
(Mean = 45.25, S.D. = 13.52)		(Mean = 10.92, S.D.=10.20)	
**Marital status**		**Relationship to older adults**	
Single	69 (21.0)	Children	233 (71.0)
Married	218 (66.5)	Grandchildren	51 (15.6)
Widowed/divorced/separated	41 (12.5)	Others (sisters/brothers)	44 (13.4)
**Education level**		**Age of older adults (years)**	
Illiterate	20 (6.2)	60–69	123 (37.5)
Primary school	107 (32.6)	70–79	134 (40.9)
Junior high school	47 (14.3)	≥80	71 (21.6)
High school/equivalent	112 (34.1)	(Mean = 72.32, S.D. =7.87)	
Diploma/equivalent	16 (4.9)	**Chronic health conditions in older adults**	
Bachelor’s degree/higher	26 (7.9)	No	131 (39.9)
**Current occupation**		Yes	197 (60.1)
Retired	66 (21.1)	**Physical impairments in older adults**	
Government officer	10 (3.0)	No	278 (84.8)
Housework	11 (3.4)	Yes	50 (15.2)
Employee	138 (42.1)	**Falls in the past year among older adults**	
Agriculturalist	60 (18.3)	No	270 (82.3)
Business	33 (10.1)	Yes	58 (17.7)
Others (freelance)	10 (3.0)	**Dwelling of older adults**	
		1-floor house	108 (32.9)
		1-floor house with a basement	81 (24.7)
		2-floor house	139 (42.4)

### Knowledge about home environmental safety management, perceived susceptibility to falls among older adults, perceived severity of falls among older adults, perceived self-efficacy of home environmental safety management, perceived outcome of home environmental safety management, and home environmental safety management

Overall, 41.8% of participants had an average level of knowledge regarding home environmental safety management, with a mean of 7.03 (S.D.= 1.51). However, the levels of knowledge regarding perceived susceptibility to falls, perceived severity of falls among older adults, perceived self-efficacy, perceived outcome, and home environmental safety management of participants were high (53.4%, 56.4%, 86.3%, 89.6%, and 60.4%, respectively), with means of 3.69 (S.D.= 0.42), 3.74 (S.D.= 0.42), 4.04 (S.D.= 0.47), 4.10 (S.D.= 0.43), and 15.48 (S.D. = 3.91), respectively (Table 2).

**Table 2:** Overall knowledge and knowledge of perceived susceptibility, perceived severity, perceived self-efficacy, perceived outcome, and home environmental safety management distributed by levels (n = 328)

Variables	n (%)
**Knowledge about home environmental safety management**	
Low (scores of 0–5)	124 (37.8)
Average (scores of 6–7)	137 (41.8)
High (scores ≥ 8)	67 (21.4)
(Mean = 7.03, S.D. = 1.51)	
**Perceived susceptibility to falls among older adults**	
Average (2.34–3.67)	153 (46.6)
High (3.68-5.00)	175 (53.4)
(Mean = 3.69, S.D. = 0.42)	
**Perceived severity of falls among older adults**	
Average (2.34–3.67)	143 (43.6)
High (3.68-5.00)	185 (56.4)
(Mean = 3.74, S.D. = 0.42)	
**Perceived self-efficacy of home environmental safety management**	
Average (2.34–3.67)	45 (13.7)
High (3.68-5.00)	283 (86.3)
(Mean = 4.04, S.D. = 0.47)	
**Perceived outcome of home environmental safety management**	
Average (2.34–3.67)	34 (10.4)
High (3.68-5.00)	294 (89.6)
(Mean = 4.10, S.D.= 0.43)	
**Home environmental safety management**	
Low (1.00-2.33)	2 (0.6)
Average (2.34–3.67)	128 (39.0)
High (3.68-5.00)	198 (60.4)
(Mean = 15.48, S.D. = 3.91)	

### Factors affecting home environmental safety management for fall prevention for older adults

The results revealed that participants who had higher mean scores for perceived severity, perceived self-efficacy, and perceived outcome tended to have better home environmental safety management than those with lower mean scores (β = 0.323, 0.311, and 0.142, respectively). Similarly, participants who had fallen in the past year were more likely to have better home environmental safety management than those without falls in the family (β = 0.217). However, participants who were grandchildren of older adults had lower mean scores for home environmental safety management than those who were children of older adults (β = -0.143). Last, participants who were widowed, divorced, or separated seemed to have lower mean scores for home environmental safety management than those who were married (β = -0.096). All of these significant factors explained 35.1% of home environmental safety management for fall prevention for older adults (adjusted R square = 0.351) (Table 3).

**Table 3:** Factors affecting home environmental safety management for fall prevention for older adults (n = 328)

Independent variables	b	Beta (β)	p value
Perceived severity	3.023	0.322	< 0.001
Perceived self-efficacy	2.580	0.311	< 0.001
Falls in the past year (Ref.* = no)	2.223	0.217	< 0.001
Grandchildren of older adults (Ref.* = children of older adults)	-1.524	-0.143	0.002
Perceived outcome	1.299	0.142	0.024
Widowed/divorced/separated (Ref.* = married)	-1.132	-0.096	0.035

Constant (a) = -11.004, R square = 0.363, adjusted R square = 0.351, F = 30.451, P < 0.001, Ref. * = Reference group

## Discussion

Our study proposed to determine factors that affected home environmental safety management for fall prevention for older adults. The factors that significantly affected home environmental safety management (p value < 0.05) included perceived severity, perceived self-efficacy, elderly family members having fallen in the past year, being grandchildren of older adults, perceived outcome, and being widowed, divorced or separated.

Participants who had higher mean scores for perceived severity of falls among older adults tended to have better home environmental safety management than those with lower mean scores. This finding was consistent with those of previous studies [13, 14]. These studies found that falls among older adults caused injuries, physical and mental impairment, and disability. Consequently, participants in these studies managed the home environment properly to prevent falls among older adults. If a person knows that a fall is dangerous to older adults’ health, the person can immediately manage home environment safety for them [21]. Furthermore, the perceived severity of diseases helps people to be concerned about their health behaviors to prevent diseases that can cause disability or death [22, 23].

This study indicated that participants who had higher mean scores of perceived self-efficacy were more likely to have better home environmental safety management than those who had lower mean scores. This finding was in line with that in the study by Chaisang et al. (2021) [12], which found that perceived self-efficacy was positively associated with fall prevention behavior among older adults. Hence, if a person expects that home environment safety management can prevent falls among older adults, he or she can take immediate action. In other words, perceived self-efficacy is an expectation related to the competency of a person and is a behavioral indicator. Furthermore, perceived self-efficacy is also a part of the thinking process correlated between knowledge and the expression of behaviors [24].

Our study found that participants who had fallen in the past year potentially managed a better home environment than those who had not. Our finding was consistent with that of a previous study [11], which indicated that if older adults had a history of falls in the past, they would manage a better home environment to prevent falls and injuries in the future. This might be because participants were aware that if older adults fall in the future, it might be dangerous to their health. In addition, most older adults had chronic health conditions and physical impairment. These factors caused more complications after falling [9, 25].

The present study revealed that grandchildren of older adults were less likely to manage home environmental safety than children of older adults. This finding was consistent with that of the study by Kortawat et al. (2018) [26], which reported that older adults who lived with their children felt more comfortable and safer since their children cared for them well in all aspects. These included daily expenditures, medical expenses, food consumption, personal hygiene, physical and mental health, and environmental management [3]. Additionally, children of older adults might have close relationships and close bonds with their parents. Grandchildren of older adults may not care for their grandparents well since they might have limited knowledge about the perceived severity of falls and lower self-efficacy than children of older adults. As a result, children of older adults play an important role in decision-making and planning for home environmental management for preventing their parents from falling [27].

Our results found that participants who had a higher mean score for perceived outcome could manage the home environment better than those with a lower mean score. This finding was the same as that in the study by Boonlue (2018) [28], which indicated that perceived outcome was positively associated with prevention behaviors. That recent study was similar to another study [29], which found that the perceived outcome of practice was related to self-care behaviors. In other words, people who have good health status tended to have better perceived outcomes than those who have poor health status. Thus, if a person has a good perceived outcome of home environmental safety management, he or she can take measures to prevent falls among older adults. This finding is in line with protection motivation theory [30].

Last, participants who were married tended to have better home environmental safety management than those who were widowed, divorced or separated. This finding was similar to that in the study by Kortawat et al. (2018) [26], which showed that married individuals had better perceptions of home environmental management for older adults. This means that married individuals who live with their elderly parents have greater opportunities to make decisions for home environmental safety management together with their parents. Therefore, married people and family members make decisions and manage better home environments to keep all family members safe, especially older adults [31].

Our study collected data from family members who looked after older adults in families. Family members play an important role in decision-making in all aspects for their elderly parents in Thai culture. These are the strengths of our study. The limitations that might have affected the results were the other factors that we did not explore, such as social support. This factor might be associated with home environmental safety management for fall prevention for older adults in the Thai social context.

## Conclusion and suggestions

Our study revealed that factors affecting home environmental safety management for fall prevention for older adults involved perceived severity of falls among older adults, perceived self-efficacy of home environmental safety management, falls within the past year, grandchildren of older adults, perceived outcome of home environmental safety management, and marital status. As a result, health care providers should educate individuals on the perception of severity and self-efficacy for those who take care of older adults in families since these two factors showed the highest influence on home environmental safety management. The focus should be on families with a history of falls among older adults. Furthermore, grandchildren of older adults and those who are widowed, divorced or separated should also be motivated to play an important role in home environment safety management. This includes education on the perceived outcome of home environmental safety management to reduce the risk of falls among older adults. Further studies should include social support to determine whether it influences home environmental safety management for fall prevention for older adults in northern Thailand.

## Data Availability

The dataset of this study is strictly protected under the terms of the Naresuan University Ethical Committee for Dissemination. Furthermore, Thailand’s Official Information Act 1997 prohibits the release of identifiable data relating to public bodies. Due to these limitations, interested parties may contact the secretary of the Naresuan University Research Ethics Committee.
